# Determination of COVID-19 Vaccine Hesitancy Among University Students

**DOI:** 10.7759/cureus.17283

**Published:** 2021-08-18

**Authors:** Waliya Sadaqat, Shanzay Habib, Ambreen Tauseef, Sheharyar Akhtar, Meryum Hayat, Syeda A Shujaat, Amina Mahmood

**Affiliations:** 1 Medicine, CMH Lahore Medical College and Institute of Dentistry, Lahore, PAK; 2 Physiology, CMH Lahore Medical College and Institute of Dentistry, Lahore, PAK; 3 Artificial Intelligence (AI) Department, Afiniti Software Solutions Pvt. Ltd., Lahore, PAK

**Keywords:** covid-19, vaccine, hesitancy, university students, curriculum

## Abstract

Introduction

With the sudden outbreak of severe acute respiratory syndrome coronavirus 2 (SARS-COV-2), vaccines appear to be the most efficient measure in combating spread. However, vaccines are only effective if a community collectively uptakes vaccination. This approach is growing increasingly difficult with the emergence of ‘Vaccine Hesitancy.’ This paper aims to determine the association between university curricula and the degree of hesitancy for the COVID-19 vaccine.

Methods

The online questionnaire assessed demographic data, prior knowledge of vaccines, attitude towards COVID-19 vaccines using an adapted version of the WHO Strategic Advisory Group of Experts (SAGE) Working Group’s Vaccine Hesitancy Survey (VHS) and factors likely to motivate vaccine uptake. By using binary scoring, the degree of hesitancy among students was determined. Exploratory Factor Analysis (EFA) on VHS revealed underlying causes of hesitancy. To analyze the dependence between hesitancy and curriculum, a chi-squared test was conducted.

Results

Medical students scored higher for prior knowledge of vaccines (M = 3.54) as opposed to non-medical students (M = 3.49). Medical students responded favorably to COVID-19 vaccines with only 1.37% showing hesitancy for all nine items of VHS, compared to 2.55% of non-medical students. EFA produced three subscales within the VHS: lack of confidence, risk factor concern, and misinformation. The lack of confidence factor accounted for 65% of the data obtained. The chi-square test solidified that vaccine hesitancy is dependent on curriculum.

Conclusion

The majority of non-medical students showed hesitancy towards obtaining COVID-19 vaccines compared to medical students who were more willing, largely owing to their knowledge and understanding of vaccines.

## Introduction

With the sudden outbreak of severe acute respiratory syndrome coronavirus 2 (SARS-COV-2), vaccines appear to be the most efficient measure of controlling the pandemic, along with wearing masks and social distancing. However, vaccines are only effective if 70% of the community collectively uptakes vaccination in order to attain herd immunity [1]. However, eradication of coronavirus disease 2019 (COVID-19) through herd immunity is growing increasingly difficult with the emergence of a phenomenon termed ‘Vaccine Hesitancy.’ The WHO Strategic Advisory Group of Experts (SAGE) Working Group describes vaccine hesitancy as “delay in acceptance or refusal of vaccines despite availability of vaccine services,” and places vaccine-hesitant individuals on a “continuum ranging from total acceptance to complete refusal” [2].

Pakistan is one of the two countries with consistent barriers preventing vaccination and the eradication of polio as reported by the Global Polio Eradication Initiative [3]. In the past, renowned political commentators, religious clerics, and journalists have made various exaggerated statements that may spark negative perceptions towards vaccines, particularly COVID-19 vaccines [4]. Despite scientific reports stating there is no evidence against adverse events following immunization (AEFI), the public has remained dissatisfied [5]. There is an inconsistency between scientific evidence and understanding of vaccine safety [6].

Medical students are future health care providers who will eventually be entrusted with counseling vaccine-hesitant individuals in the future, which is only possible if the importance of vaccines is highlighted during the course of their education. Generally, non-medical students are taught a plethora of courses throughout their undergraduate program; however, the vaccine-related syllabus is often excluded from their curricula, which may affect how they perceive vaccines. It is critical to accomplish a high COVID-19 vaccine coverage rate among young adults, as they are at a higher risk of becoming infected and transmitting the novel virus, under the false assumption that they are invulnerable to the infection [7].

There is a dire need to understand the vaccination perception of students and the extent of hesitancy in order to achieve the high uptake rates required for herd immunity. Moreover, the need to assess the impact of educational curricula on students’ attitudes towards vaccinations is equally imperative so that reformed educational strategies can be implemented to tackle any misleading notions they hold [8]. Additionally, it is critical to determine possible incentives that will increase vaccination so the government may employ efficient vaccination programs.

## Materials and methods

Study design, sampling, and data collection 

This cross-sectional qualitative study was carried out using an online anonymous questionnaire on university students across Pakistan. The sample size calculated was 400 and the total duration of this study period was six months. The questionnaire was shared on the college forums and discussion groups as well as on social networking websites, such as Facebook and WhatsApp. University students between the ages of 18 and 25 years, who had not yet obtained COVID-19 vaccination, were eligible to partake in this study.

Measures

Electronic informed consent was obtained to maintain the anonymity of the participants. No information was disclosed or stored. Ethical approval was obtained from the institutional review board (IRB) of CMH Lahore Medical College and Institute of Dentistry (Case #.594 /ERC/CMH/LMC).

The questionnaire included the following components: 1) Demographic data, 2) Prior knowledge of vaccines, 3) Vaccine Hesitancy Survey, 4) Incentives that may increase vaccination uptake.

Prior knowledge of the COVID-19 vaccine was assessed through four true/false statements [9]. Attitude towards COVID-19 vaccines was assessed by adapting the 10-item Vaccine Hesitancy Scale (VHS) developed by the SAGE Working Group [2]. The statements were modified to a more generic version, e.g. ‘‘Childhood vaccines are important for my child’s health” to ‘‘Vaccines are important for my health,” an approach previously adopted in a study by Luyten et al. [10]. One question, “Generally, I do what my doctor or healthcare provider recommends about vaccines,” was dropped as the survey already targets future doctors/healthcare providers. As vaccine hesitancy includes both refusal and delay in acceptance, the responses were assessed on a five-point Likert scale. Lastly, participants were asked to choose from a list of statements that could possibly increase or further increase vaccine uptake [1]. Data collected were analyzed using R software, version 4.0.5 (R Foundation for Statistical Computing, Vienna, Austria).

## Results

Demography

The survey distributed gathered 418 responses, out of which 415 of the respondents were willing to partake in the survey. Out of these 415 respondents, 219 (52.8%) were medical students and 196 (47.2%) were non-medical students. 282 (68%) of the respondents were females and 133 (32%) were males.

Prior knowledge and hesitancy scoring

Each respondent was scored on a scale of 0 to 4 for their prior knowledge of COVID-19 vaccines based on the number of statements they got correct. The mean scores for medical and non-medical students were 3.54 and 3.49, respectively.

**Figure 1:**
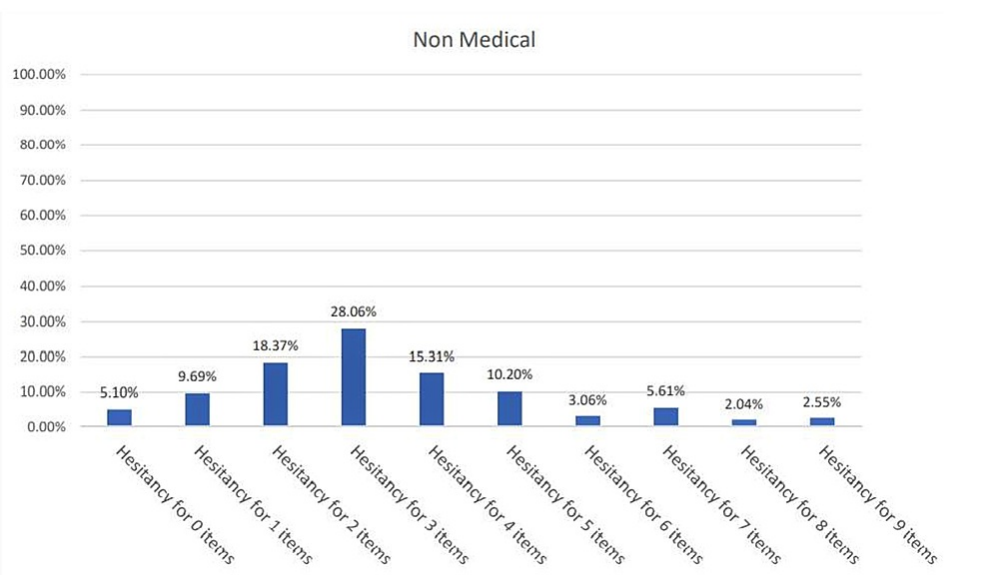
Percentage (%) of hesitant responses per VHS item for non-medical students VHS: Vaccine Hesitancy Survey

**Figure 2:**
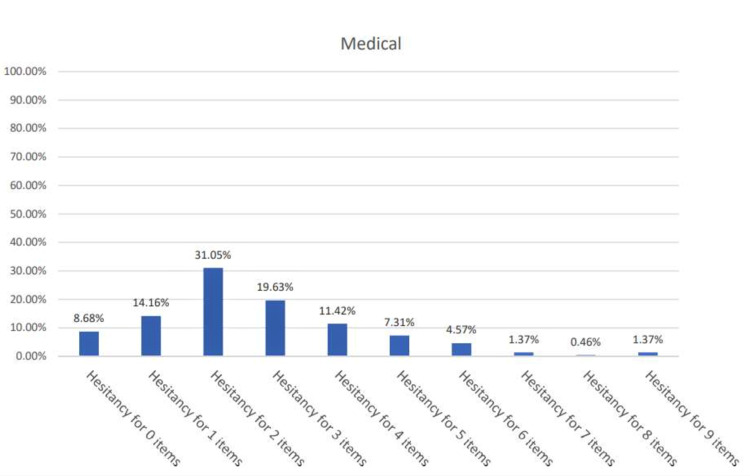
Percentage (%) of hesitant responses per VHS item for medical students VHS: Vaccine Hesitancy Survey

In order to assess which population group showed a greater degree of hesitancy, a scoring system was employed. The VHS Likert scale was converted to binary responses by scoring options 1, 2, and 3 as ‘hesitant’ and options 4 and 5 as ‘not hesitant.’ The percentage of participants hesitant for n number of items was found for each curriculum as shown in Figures 1-2.

The percentage of ‘hesitant’ respondents was greater in the non-medical students, with 2.55% showing hesitancy for all nine items as compared to only 1.37% of the medical students.

Vaccine Hesitancy Survey analysis

The Vaccine Hesitancy Survey was analyzed using exploratory factor analysis (EFA). Eight outliers were found in the data using the Mahalanobis distance method, which were then excluded from consideration. The resulting sample was deemed adequate for EFA using the Kaiser-Meyer-Olkin factor (.86) and Bartlett’s test (P <.001). Prior to performing the EFA, parallel analysis was run on the data to determine how many factors were adequate to maximize the loading scores of the items. Three factors were found to be significant.

One of the items was dropped due to a lack of loading on any of the three factors. The factor loadings and the chi-squared test for each item with the curriculum is presented in Table 1. The cutoff loading score selected was 0.3.

**Table 1:** Loading scores, uniqueness, and communality of each item * P-values ≤ 0.05 are significant.

Items	Lack of confidence (Factor 1)	Risk factor concern (Factor 2)	Misinformation (Factor 3)	Adjusted P-value of Χ^2^- test with curriculum
“Vaccines are important for my health”	.81	.19	.15	.000903
“Vaccines are effective”	.71	.10	.32	.00538
“Vaccines are important for the health of others in my community”	.87	.06	.12	.014
“All vaccines offered by the government program in my community are beneficial”	.52	.09	.39	.000345
“New vaccines carry more risks than older vaccines”	.05	.61	.08	.038
“The information I receive from the vaccine program is reliable and trustworthy”	.28	.16	.73	.000258
“Getting vaccines is a good way to protect myself from the disease”	.73	.10	.22	.00718
“I am concerned about serious adverse effects of vaccines”	-.09	-.43	-.06	.37

The EFA accounts for 54% of the variation in the responses, 65% of which is explained by factor 1, named ‘Lack of Confidence’ factor, 20% explained by factor 2, named ‘Risk Concerns’ and 15% explained by factor 3, named ‘Misinformation.’ The reliability and fit of the EFA were checked using multiple indices (Tucker-Lewis index = .996, RMSEA = .019, comparative fit index = .999, df corrected RMSR = .03), which all responded favorably. In addition, the chi-squared metric with the null hypothesis that three factors were sufficient to explain the variation in the data also suggested a good fit, with P=.325.

A chi-squared test was applied on each item and the curriculum was studied at a 5% significance level, to confirm if the hesitancy of students is dependent on curriculum. As shown in Table 1, all the items loaded on the ‘Lack of Confidence’ factor are strongly dependent on the curriculum.

Analyzing motivational roots

**Figure 3:**
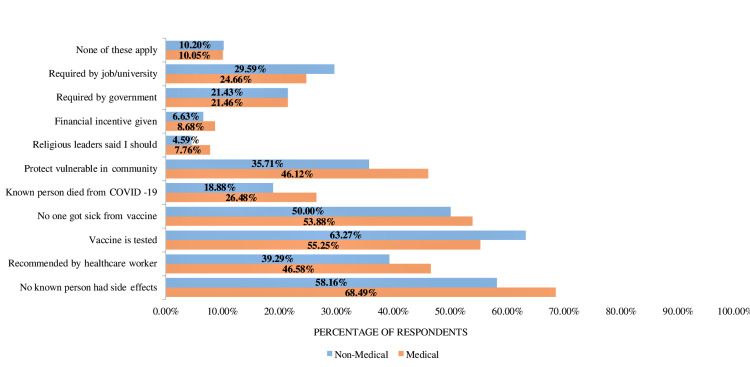
Percentage of university students likely to obtain vaccines according to motivational factors

Figure 3 illustrates that 63.27% (124) of non-medical students would be more inclined to get the vaccine if they were convinced that the vaccines had been rigorously tested, whereas 68.49% (150) of medical students would further increase vaccine uptake if they saw that enough people, including their family members, did not experience any side-effects from the vaccine. With an average of 25.6 students (6.18%), incentivization provided through religious leaders of the community was least likely to encourage vaccine uptake for both groups (Figure 3).

## Discussion

Overall, medical students show a greater degree of acceptance toward COVID-19 vaccines. These results warrant attention as previously Barello et al.’s study in Italy found no significant differences in the responses of the two groups [8]. The difference could be attributed to the fact that their study was conducted in the earlier days of the COVID-19 pandemic when there was still ongoing research and ambiguity surrounding the novel virus and vaccines that were undergoing clinical trials.

After running an exploratory factor analysis on VHS, the factors found accounted for 54% of the variation. Domek et al. [11] reported 76% of variation explained by two underlying constructs “Confidence” and “Complacency/Risks, Shapiro et al. [12] reported 67% of variation explained by two underlying constructs, “Lack of confidence” and “Risks,” and lastly, Luyten et al. [10] reported 72% of variation explained by two underlying constructs “Lack of confidence” and “Risks.” The difference in variation may be because the responses collected were greater than 1000 in all three past pieces of research.

It was found that out of the three factors produced from EFA, “Lack of Confidence” had the highest mean score (M=4.409, SD=.622) and, therefore, is the most significant factor in influencing hesitancy. A finding observed was that not all the questions that loaded onto factor ‘Risk Concerns’ were significantly dependent on curricula, which is understandable since it is normal for anyone to be worried about potential adverse effects regardless of educational background. Similarly observed by Reno et al. [13], despite participants’ age, gender, income, socio-economic or educational background, safety concerns were the key reason for hesitancy.

Medical students showed a greater prior knowledge of vaccines as compared to non-medical students, further strengthening the presumption that they are better informed about the advancements in the health sector. Previous research has shown there is increased hesitancy in people with lower awareness levels of vaccines, which falls in line with our results [14-15]. Higher levels of knowledge are associated with an increase in preventative behavior such as following standard operating procedures (SOPs) and getting vaccinated [16]. Health-related topics are neither prioritized nor addressed in non-medical curricula, which may create a sense of distrust and reluctance as these groups of individuals lack relevant information [17]. Multidisciplinary education and vaccination seminars should be integrated into all university curricula that should be updated regularly in order to keep up with the new advancements in vaccines [18]. It is worth noting that medical students did not score a perfect 4 as would be expected from them. Future healthcare professionals must address their own unanswered queries and doubts regarding vaccines before they can bridge the knowledge gap amongst the general public [19].

Incentives that would increase vaccination uptake were more or less the same for both groups. By understanding the motivational roots, appropriate public intervention and pro-vaccination projects can be introduced with greater efficiency. As stated by Saied et al. [15], hesitant individuals are expected to change attitudes if provided with information from reliable and trustworthy sources regarding a vaccine’s efficacy and safety.

The strength of the current study is that it not only compares vaccine perception held amongst university students but also explores possible incentives to increase acceptance; research that is first of its type in Pakistan. The current study took place after vaccines were introduced in Pakistan. This allowed us to realistically analyze and assess university students’ first impressions of the COVID-19 vaccine. Some limitations have been observed in this study. “Vaccine Hesitancy is influenced by factors such as confidence, complacency, and convenience," as acknowledged in the SAGE Working Group’s ‘3 C’s Model’ [2]. However, VHS primarily focuses upon confidence in vaccines, demonstrated by the three constructs derived from EFA, and does not cover other possible determinants. Certainly, there are other motivations that may cause an individual to show reluctance that needs to be addressed and incorporated in order to devise a standard and validated tool to assess vaccine hesitancy [20]. Moreover, as observed by Larson et al. [21], ongoing monitoring is required due to the dynamic nature of vaccine hesitancy, especially since COVID-19 awareness and pro-vaccination education programs are slowly being introduced to the public. Additionally, the sample size obtained was small, which primarily focused on respondent’s educational background, further research is required to understand other socio-demographic factors such as income, rural/urban background, etc.

## Conclusions

In conclusion, 95% of non-medical students showed some degree of hesitancy towards COVID-19 vaccines. Lack of confidence was the major contributor to hesitancy. Overall, there was a significant correlation between vaccine hesitancy and curriculum. Medical students were more likely to obtain vaccines owing to their literacy in health education. Multidisciplinary education, pro-vaccination awareness campaigns, and health seminars must be introduced by government authorities to promote a positive attitude towards vaccines.
